# Assessment of arrhythmias and heart rate response in healthy adolescents performing face immersion and body submersion in ice‐cold water

**DOI:** 10.14814/phy2.70430

**Published:** 2025-07-07

**Authors:** Anna Lundström, Marcus Karlsson, Annika Rydberg, Urban Wiklund

**Affiliations:** ^1^ Department of Clinical Sciences Umeå University Umeå Sweden; ^2^ Department of Diagnostics and Intervention Biomedical Engineering and Radiation Physics, Umeå University Umeå Sweden

**Keywords:** adolescents, arrhythmias, cold‐water immersion, face immersion, heart rate

## Abstract

As cold‐water immersion becomes more popular and accessible, it is important to explore potential risks. This study examines the cardiac autonomic response and arrhythmia occurrence in healthy adolescents during face and body immersion. Healthy ninth‐grade students, aged 15–16 years, were recruited to perform face immersion (FI) in 10°C water and body immersion in 2°C water (IWI). Electrocardiograms (ECGs) were continuously recorded, and the heart rate (HR) response and occurrence of arrhythmias were assessed. Among the 54 individuals performing FI, six had supraventricular extrasystoles, and two had ventricular bigeminy. Among the 20 performing IWI, four had supraventricular extrasystoles. The HR response was more pronounced during FI compared to IWI (*p* < 0.001). During both FI and IWI, girls showed initially higher HR and more pronounced HR reduction than boys, but there were no significant sex differences (*p* = 0.26). During the first 30 seconds of IWI, boys maintained a steady HR (*p* = 0.176), while girls experienced a near‐linear reduction (*p* = 0.009). This study indicates a low risk of severe arrhythmias when briefly immersing the body in ice‐cold water in healthy adolescents. However, the risk could increase if combined with face submersion and apnea.

## 1 INTRODUCTION

Body immersion into cold water has become an increasingly popular activity in Northern Scandinavia. In Sweden, rather than swimming actively in the cold water, the usual practice involves immersing the body into the cold water through a small hole made in the frozen lake or sea. This activity has received praise from those who practice it and has become a popular topic in the mainstream press, with claims of numerous health benefits such as boost immune system, improved mental health and reduced stress (Knechtle et al., 2020). However, winter swimming, with its exposure to ice‐cold water, is a physically demanding activity that may pose health risks, especially for individuals unaccustomed to bathing or swimming in cold water (Manolis et al., 2019).

The impact of sudden exposure to cold water immersion triggers the activation of the neurogenically mediated cold shock response, which induces uncontrollable heavy breathing followed by hyperventilation (Tipton, 1989). Additionally, there is simultaneous vasoconstriction, accompanied by a rapid elevation in heart rate, blood pressure, and cardiac output. This reaction primarily involves the sympathetic nervous system (SNS), initiated by peripheral cold receptors. The reaction peaks after approximately 30 s, and adaptation takes about 2 min, resulting in the ability to regain control of breathing. This stage is acknowledged as the most dangerous period, contributing to the majority of deaths resulting from immersion in cold water (Tipton et al., 1999). Death can result either due to drowning, as even a small amount of aspirated water can initiate the drowning process due to the loss of breathing control caused by initial cold shock, or due to cardiac arrhythmias (Asplund & Creswell, 2016). The risk of developing arrhythmias increases if both branches of the autonomic nervous systems (ANS) are activated simultaneously (Shattock & Tipton, 2012). If water enters the orofacial area or if the head is submerged under water, resulting in apnea, the diving reflex is triggered. Subsequently, the parasympathetic nervous system (PNS) is activated through the vagal nerve, resulting in bradycardia and a decreased workload on the heart. This dual activation of the autonomic nervous systems (ANS) two branches can lead to an autonomic conflict, which increases the risk of potential life‐threatening arrhythmias during swimming (Shattock & Tipton, 2012).

Since cold water immersion has gained popularity and thereby accessibility, there is an increasing number of individuals from various age groups partaking in this activity. This highlights the importance of examining the potential risks associated with it. The objective of this study was to examine the cardiac autonomic response and the occurrence of arrhythmias in healthy adolescents during face immersion and body immersion in ice‐cold water.

## 2 MATERIALS AND METHODS

### 2.1 Study cohort

In some schools in this region, ice‐bathing is a part of the physical education during the ninth year at school. Therefore, healthy ninth‐grade students, aged 15–16 years, were recruited for participation. Information was distributed to all pupils in five school classes, followed by a verbal presentation of the study, allowing the children to ask questions. Informed written consent was obtained from the legal guardians of the participants. Prior to participation, participants completed a questionnaire to provide information on clinical details, including date of birth, gender, existing medical conditions, and ongoing medical treatment.

### 2.2 Water provocations

The water provocations were divided into two separate sessions, and both occurred either as a part of the participants' physical education class or in direct association with it. Each of the sessions followed its own protocol for the water provocations. The face immersion (FI) took place indoors and included immersing the face in 10°C water (Foster & Sheel, 2005; Jay et al., 2007). The temperature was chosen to evoke a pronounced diving reflex (Gooden, 1994). The aimed duration was 25 s, but not all participants managed to reach this goal. The body immersion in ice‐cold water (ice‐water immersion, IWI) took place outdoors during the winter (February) at a lake near Umeå, a city located in the northern part of Sweden. At the start of the IWI procedure, the ECG recorder was attached indoors. The participants wore clothes suitable for indoor conditions. They then proceeded to walk approximately 200 meters out onto the frozen lake, where a 1.5 × 1.5 meters opening had been prepared in the ice. With a water temperature of 2°C, participants, equipped with ice prods, a backpack, and a waist‐secured rope for precautionary measures, immersed themselves in the water. The whole body, but not the head, was immersed. The immersion time was documented. To avoid hyperventilation, participants were instructed to take deep breaths, and then they were required to answer a short, straightforward question, such as “Which is the capital of France?,” before exiting the cold water. The guideline was that participants should be in the cold water for a maximum of 30–60 s. They then used the ice prods to pull themselves out of the water, and the exit time was documented.

### 2.3 Electrocardiogram (ECG)

Continuous ECG monitoring was conducted using the Actiwave‐Cardio monitor (CamNtech, Cambridge, UK), which is a waterproof single‐channel ECG recorder. Additionally, this device monitors and stores body movement and position through a tri‐axial accelerometer. Both the monitor and the two electrodes were covered with water‐resistant adhesive plasters in order to minimize disturbances. The ECG monitor samples ECG data at a rate of 500 Hz, and a custom‐made software automatically detects heartbeats. Errors in the detection were manually corrected, and instances of arrhythmic beats were noted.

Additionally, during IWI the participants were also equipped with a Garmin HRM‐swim heart rate monitor chest strap (Garmin, Lenexa, Kansas). The chest strap was connected to a Fenix 6 Garmin fitness and sports watch (Garmin, Lenexa, Kansas), which recorded one heart rate (HR) value per second. The data from the Garmin monitor was only used as a complement during the error correction of the IWI recordings. Upon reviewing recordings from FI, it was found that the Garmin monitor had not successfully captured the more rapid changes in HR that occurred during FI, making the data unsuitable for analysis.

### 2.4 Heart rate during face immersion

There was a fast decrease in HR after the start of FI that lasted until they aborted the immersion, where the maximum and minimum HR occurred at different time points in each subject during FI. Therefore, the following indices were determined. Baseline HR was calculated as the average during the last 5 s before the start of FI. The maximum and minimum HR were determined during the immersion, and the decrease in HR was defined as the difference between them. The duration was defined as the time between the start of FI and the end of the immersion.

### 2.5 Heart rate during ice‐water immersion

The HR response had a less pronounced reaction with variable and slower changes during IWI as compared to FI. To reduce the impact of disturbances in the ECG due to intermittent muscular activity during IWI, HR was calculated as consecutive 5‐s averages from 5 s before the start of IWI to 30 s after the immersion. Following that, as the subjects started to exit the water, the ECG could not be analyzed due to excessive interference from movements and muscular activity.

### 2.6 Statistical analyses

Descriptive data for continuous variables is presented as means ± SD. The Mann–Whitney U test was used to assess differences in HR between the two sexes during FI and IWI. The Friedman test was used to examine differences in HR across multiple time points during IWI. The selected time points included the initiation of immersion and subsequent 5‐s intervals throughout the immersion period, reaching up to 30 s. Post hoc analysis was conducted with Wilcoxon signed‐rank tests. The pairwise comparison between FI and IWI was conducted with Wilcoxon signed‐rank tests.

The overall HR response during each immersion was presented in the figures as group averages, where standard errors of the mean (SEM) values were used as an estimate of the uncertainty in the response. This was determined by converting the original beat‐to‐beat HR data to synchronized and equidistantly sampled data by cubic spline interpolation and resampling at 4.8 Hz. The group averages were smoothed by calculating one‐second moving averages. IBM SPSS v28 (IBM Corp., Armonk, NY, USA) and Matlab R2024b (Mathworks Inc., Natick, MA, USA) were used for all analyses.

### 2.7 Ethical aspects

The study was approved by the Regional Ethical Board in Umeå, Sweden (Dnr 2017–145‐31 M) and was conducted in accordance with the Declaration of Helsinki. All participants, as well as their legal guardians, provided written informed consent before participating in the study.

## 3 RESULTS

### 3.1 Study cohort

Fifty‐five individuals performed FI, but one was excluded due to a duration less than 10 seconds. Among these 54 individuals, 23 also participated in IWI. However, two were excluded from the IWI analysis due to significant ECG disturbances, and one was excluded for having a duration in the water that was considered too short. This resulted in the inclusion of 20 individuals in the IWI analysis.

### 3.2 Arrhythmias

Among the remaining 54 individuals who performed FI, five had occasional supraventricular extrasystoles, and one experienced supraventricular extrasystoles at regular intervals during FI. One individual had ventricular bigeminy at the end of FI, and one had ventricular bigeminy after completing FI.

Among the remaining 20 who performed IWI, three had occasional supraventricular extrasystoles and one experienced supraventricular extrasystoles at regular intervals.

### 3.3 Face immersion

**FIGURE 1:**
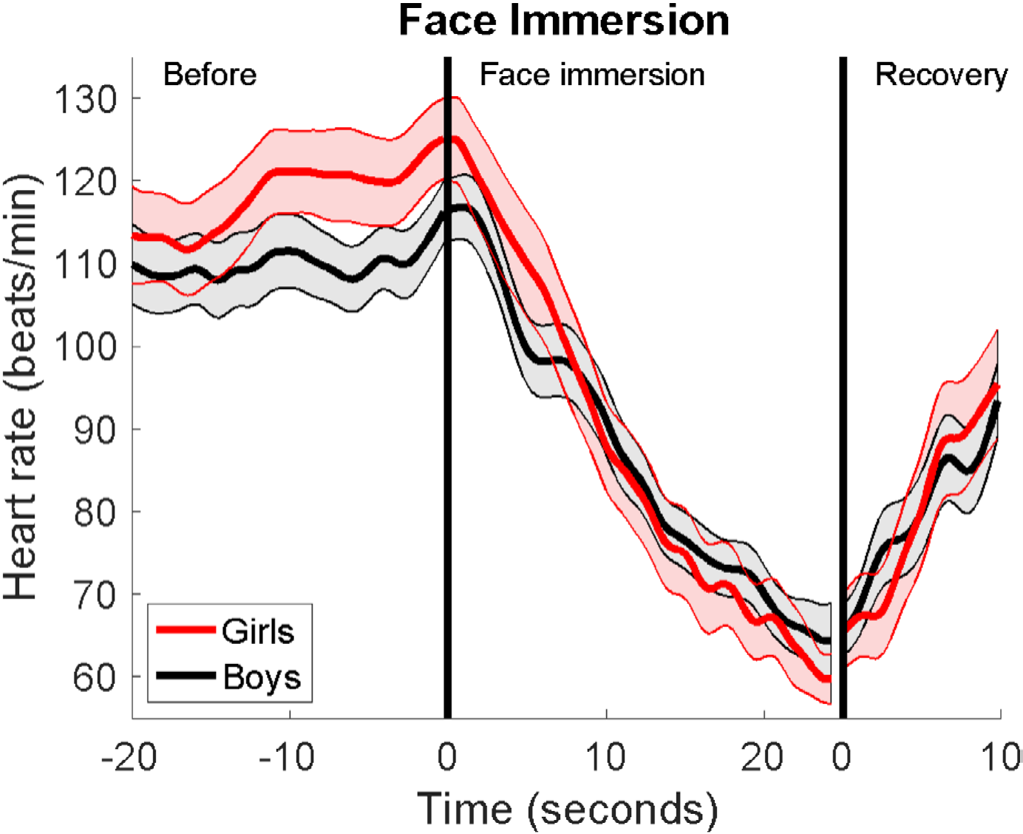
Heart rate reaction in boys and girls during face immersion. Data is presented as means and standard error of the mean (SEM). *N* = 21 for girls. *N* = 33 for boys.

A total of 54 adolescents performed face immersion with long enough exposure (mean duration 25.7 ± 5.3 s, range 10.7–35.1 s). Descriptive statistics for the entire group are presented in Table 1. Across the entire cohort, the FI induced a mean reduction in the HR by 68 bpm. Although the reduction was more pronounced in girls, the difference was not statistically significant (Figure 1). Overall, the analysis of HR reaction between girls and boys revealed no significant differences in event duration or in any of the HR indices (Table 1).

**TABLE 1:** Heart rate reaction during face immersion.

	The entire cohort (*N* = 54)	Girls (*N* = 21)	Boys (*N* = 33)	*p* value girls versus boys
Duration (s)	25.7 (5.3)	26.0 (4.9)	25.5 (5.6)	0.72
Baseline HR	116 (22)	122 (23)	112 (22)	0.10
Max HR	125 (20)	129 (22)	124 (19)	0.39
Min HR	58 (16)	57 (19)	58 (15)	0.60
Decrease	68 (19)	71 (20)	65 (18)	0.26

*Note*: HR values are given in beats per minute (bpm). Data are presented as mean and standard deviation (SD). Baseline HR represents the HR value at the start of FI. Decrease, the difference between Max and Min HR; *N*, number of subjects; HR, heart rate.

### 3.4 Ice‐water immersion

**FIGURE 2:**
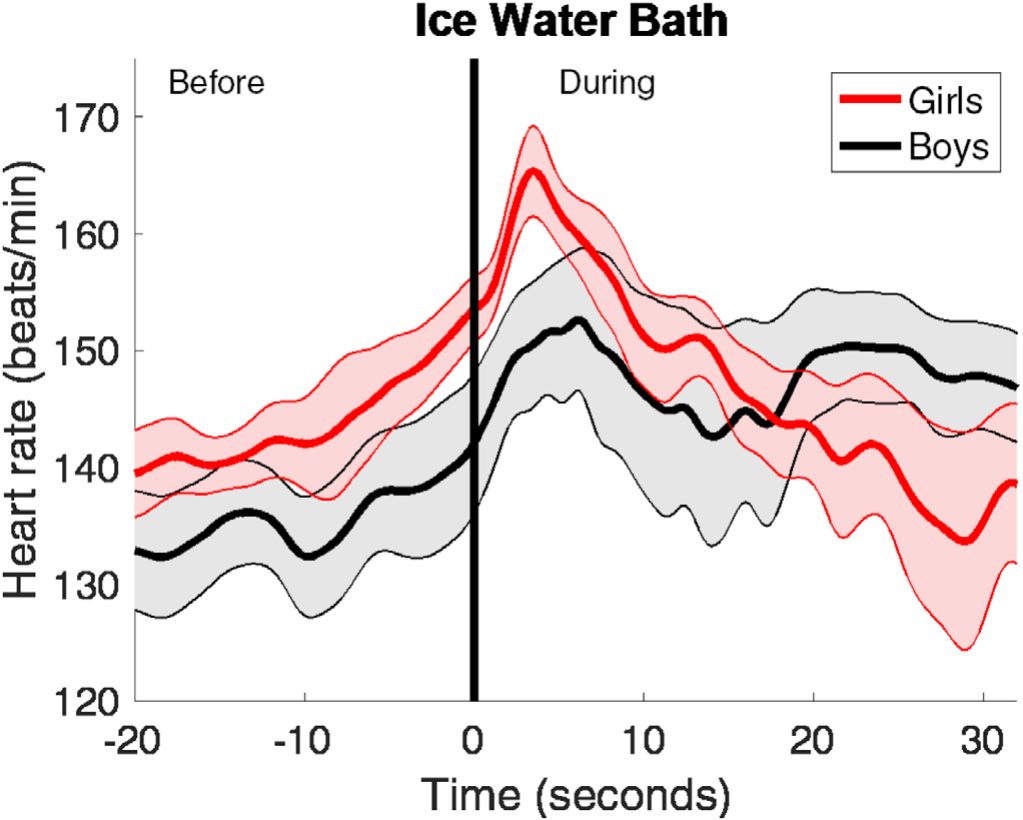
Heart rate reaction in boys and girls during ice‐water immersion. Data is presented as means and standard error of the mean (SEM). *N* = 8 for girls. *N* = 10 for boys.

Among the 20 participants included in the arrhythmia analysis, two were excluded from the heart rate response analysis due to a gap of approximately 10 s in their ECG data during the first 30 s of immersion. Consequently, the analysis comprised 18 participants, of whom 8 were girls (44%). The mean duration was 42.0 ± 9.2 s, range 28.0–65.0 s. In the entire cohort, HR increased before the start and 5 s into the immersion in ice‐cold water (Table 2). Following this, the progression appeared to differ slightly between girls and boys (Figure 2). However, when comparing the HR at different time points, no significant differences were found between girls and boys (Table 2). There was no significant variation in heart rate observed in boys from start and during the initial 30 s of IWI (*p* = 0.176). In contrast, the girls presented with a significant change in HR during the same period (*p* = 0.009). The girls first increased their HR during the first 5 s of immersion from 156 to 161 bpm; after that, the HR made a marked decrease down to 137 bpm at the time point 30 s.

**TABLE 2:** Heart rate reaction during body immersion in ice‐cold water. HR values are given in beats per minute (bpm). Data are presented as mean and standard deviation (SD). N, number of subjects; HR, heart rate.

	The entire cohort (*N* = 18)	Girls (*N* = 8)	Boys (*N* = 10)	*p* value girls versus boys
HR 5 s before start	143 (15)	148 (10)	138 (17)	0.32
HR at start	150 (15)	156 (7)	145 (17)	0.24
HR after 5 s	156 (15)	161 (8)	153 (18)	0.36
HR after 10 s	148 (21)	152 (11)	144 (27)	0.57
HR after 15 s	146 (20)	147 (11)	145 (25)	0.52
HR after 20 s	147 (15)	142 (14)	151 (15)	0.20
HR after 25 s	143 (20)	136 (24)	149 (15)	0.20
HR after 30 s	143 (18)	137 (21)	148 (14)	0.24
Friedman test of within‐group changes (*p* value)		0.009	0.18	

### 3.5 Comparison between face immersion and ice‐water immersion

In a pairwise analysis involving the 18 participants who underwent both FI and IWI, there was a notable difference in HR reduction comparing the two procedures. During the initial 25 s of FI, participants showed a pronounced decrease of 59 ± 19 beats, in contrast to a more modest reduction of 7 ± 23 beats during the first 25 s of IWI (*p* < 0.001).

## 4 DISCUSSION

The focus of this study was to examine the incidence of arrhythmias and the cardiac autonomic response during face immersion and ice‐water immersion in healthy adolescents. In summary, supraventricular extrasystoles were common both during FI and IWI, whereas ventricular extrasystoles occurred only during FI. Additionally, the HR response was more pronounced during FI compared to IWI. In both FI and IWI, girls initially had a higher HR and a more pronounced reduction in HR than the boys, but these differences were not statistically significant. The HR progression during the first 30 seconds of IWI differed between boys and girls, with boys maintaining a relatively steady HR throughout the IWI, whereas girls experienced a marked and near‐linear reduction.

Both FI and IWI induce a high level of activation in the ANS. In the case of FI, this activates the diving reflex, and FI has been shown to be useful in an experimental setting as a substitute for whole‐body submersion (Shamsuzzaman et al., 2014). The diving reflex is a cardiorespiratory response to submersion under water, and is characterized by apnea, bradycardia, and peripheral vasoconstriction. Both branches of the autonomic nervous system work together to coordinate the physiological changes that occur during the diving reflex, but regarding the autonomic control over the heart the PNS dominates. Activation of the PNS helps conserve oxygen by reducing the heart rate and depending on the temperature of the water the SNS causes peripheral vasoconstriction, especially in the extremities. This constriction of blood vessels helps to redirect blood flow to vital organs like the heart and brain, further contributing to the preservation of oxygen. Furthermore, research indicates a link between the diving reflex and the trigemino‐cardiac reflex, which is triggered by mechanical stimulation in the forehead, periorbital, and nasal regions (Cornelius et al., 2010). When the face is immersed, the water in the facial area triggers this reflex, activating the trigeminal nerve. This activation results in bradycardia, hypotension, and gastric hypermobility, thereby reinforcing the diving reflex (Costalat et al., 2013).

During IWI, the participants immersed their bodies, keeping their heads above the ice‐cold water. This immediately initiates the cold shock response, the body's physiological reaction to sudden exposure to cold water, leading to a rapid activation of the SNS (Tipton et al., 2017). This activation induces an initial and involuntary gasp reflex, followed by a sensation of breathlessness and hyperventilation. Simultaneously, there is peripheral vasoconstriction, an increase in HR and blood pressure, and a release of stress hormones. These responses are an attempt to minimize heat lost and maintain oxygen delivery to the vital organs. Although there may be some physiological reactions that overlap, it's important to differentiate between the cold shock response and the diving reflex. The cold shock response is more immediate and is associated with the initial contact with cold water, while the diving reflex occurs during more prolonged submersion.

The physiological responses to water activities, particularly in cold water, can potentially trigger arrhythmias in some individuals, even if they are generally healthy (Asplund & Creswell, 2016; Tipton et al., 1994; Wierzba et al., 2011). Water activities, particularly those involving submersion and apnea, have the potential of activating both branches of the ANS simultaneously. This can alter the balance between both branches and cause conflicting inputs on the heart, thereby leading to an autonomic conflict and arrhythmias (Shattock & Tipton, 2012). In this study with a limited number of healthy adolescents, supraventricular extrasystoles were found to be common during both FI and IWI, while ventricular extrasystoles were observed exclusively during FI. Because FI in 10 degrees water will activate both the diving reflex and the cold shock response, whereas IWI will predominantly activate the cold shock response, the activation of both branches of the ANS is more pronounced during FI than IWI. This dual activation increases the risk of autonomic imbalance in the heart, elevating the likelihood of developing arrhythmias in predisposed individuals.

As expected, the participants in this study had a more pronounced reduction in HR during FI compared to IWI. A rapid decrease in HR requires a strong activation of the PNS. The diving reflex, characterized by apnea and activation of cold receptors in the face, acts as a potent mediator in facilitating this process. In contrast, there is an initial quick and deep inhalation during IWI, but this rapidly transitions into hyperventilation. Consequently, the PNS response is much weaker, resulting in a less pronounced reduction in HR. The girls in this study generally had a higher HR than boys. All participants in the study had undergone puberty, and since the heart is proportionate to body size, the female heart is typically smaller. Consequently, it pumps less blood in each heartbeat, requiring a faster HR to maintain the cardiac output (Prabhavathi et al., 2014). Even though girls had a more pronounced reduction in HR during both FI and IWI, this was not statistically significant, and this is supported by other studies showing no differences between the sexes (Cherouveim et al., 2013; Tominaga et al., 1997). In contrast to the boys, the girls experienced a significant and nearly linear decrease in heart rate during the initial 30 s of IWI. The IWI was a stressful event for all participants and the level of anxiety differed greatly between the individuals. Therefore, an anticipatory sympathetic activation was generally high in most participants, and this could probably affect the individual heart rate responses. However, there are studies indicating that girls and young women have a higher stress‐related cardiac reactivity and recovery than equally aged boys and men (Kudielka et al., 2004). This, combined with a relatively small sample size, could probably explain these diverse results between the two sexes.

Baseline heart rates were relatively high prior to each cold‐water immersion. Each recording session included approximately 20 participants, all of whom were required to complete the immersion during a scheduled school session. Consequently, ECG monitors could only be applied, and baseline measurements recorded, shortly before immersion. It is likely that heart rates were elevated at this time due to anticipatory stress or excitement related to the impending exposure to cold water.

Since body immersion in ice‐cold water is becoming a more common practice in many parts of the world, the need to investigate its potential harmful effects increases. In this study on healthy adolescents, no ventricular premature beats were observed. The circumstances in which these adolescents, fully clothed, immersed themselves in the ice‐cold water is comparable to a situation in which someone walks or tour skates on natural ice, and suddenly the ice breaks, making the person to fall into the cold water. This suggests that, under these circumstances and with a relatively short duration in the ice‐cold water (<60 s), the risk of severe arrhythmias is relatively low in this age group, provided that the individual does not have a predisposition to develop arrhythmias. However, it is important to emphasize that the risk probably is higher if the whole body including the face, is submerged into the ice‐cold water and both the diving reflex and the trigemino‐cardiac reflex is activated (Bierens et al., 2016). Therefore, when engaging in new and, in this case, extreme activities, it is always advisable to begin cautiously and avoid swimming alone.

## 5 CONCLUSION

The results presented in this study showed that the diving reflex‐mediated FI induced a more pronounced HR reduction and ventricular extrasystoles, compared with cold shock‐mediated IWI, which only triggered supraventricular extrasystoles. These findings imply that the risk of severe arrhythmias is relatively low when briefly immersing only the body in healthy adolescents without a predisposition to develop arrhythmias. However, the risk is likely higher when ice‐cold water is combined with whole‐body submersion and apnea.

## FUNDING INFORMATION

This work was supported by the Swedish Heart Lung Foundation (A.R. Grant number: 20210468), Oskarfonden (https://www.oskarfonden.se) and through a regional agreement between Umeå University and Region Västerbotten (ALF) (A.R. Grant number: RV828131).

## CONFLICT OF INTEREST STATEMENT

The authors have no conflict of interest to declare.

## ETHICS STATEMENT

The study was approved by the Regional Ethical Review Board in Umeå, Sweden.

## CONSENT

Written informed consent was obtained from all participants.

## Data Availability

Data is available upon request.
